# Robust and Fast Lithium Storage Enabled by Polypyrrole-Coated Nitrogen and Phosphorus Co-Doped Hollow Carbon Nanospheres for Lithium-Ion Capacitors

**DOI:** 10.3389/fchem.2021.760473

**Published:** 2021-09-24

**Authors:** Mengdi Zhang, Xuan Zheng, Jiawei Mu, Pengfei Liu, Wenhan Yuan, Shuli Li, Xiaobo Wang, Haiqiu Fang, Haiyan Liu, Tao Xing, Han Hu, Mingbo Wu

**Affiliations:** ^1^ State Key Laboratory of Heavy Oil Processing, Institute of New Energy, College of Chemical Engineering, China University of Petroleum (East China), Qingdao, China; ^2^ New Energy Division, ShanDong Energy Group CO., LTD., Zoucheng, China

**Keywords:** lithium-ion capacitors, anode materials, porous carbon, hollow structure, heteroatom doping, conductive polymers

## Abstract

Lithium-ion capacitors (LICs) have been proposed as an emerging technological innovation that integrates the advantages of lithium-ion batteries and supercapacitors. However, the high-power output of LICs still suffers from intractable challenges due to the sluggish reaction kinetics of battery-type anodes. Herein, polypyrrole-coated nitrogen and phosphorus co-doped hollow carbon nanospheres (NPHCS@PPy) were synthesized by a facile method and employed as anode materials for LICs. The unique hybrid architecture composed of porous hollow carbon nanospheres and PPy coating layer can expedite the mass/charge transport and enhance the structural stability during repetitive lithiation/delithiation process. The N and P dual doping plays a significant role on expanding the carbon layer spacing, enhancing electrode wettability, and increasing active sites for pseudocapacitive reactions. Benefiting from these merits, the NPHCS@PPy composite exhibits excellent lithium-storage performances including high rate capability and good cycling stability. Furthermore, a novel LIC device based on the NPHCS@PPy anode and the nitrogen-doped porous carbon cathode delivers a high energy density of 149 Wh kg^−1^ and a high power density of 22,500 W kg^−1^ as well as decent cycling stability with a capacity retention rate of 92% after 7,500 cycles. This work offers an applicable and alternative way for the development of high-performance LICs.

## Introduction

In the past decades, electrochemical energy storage devices represented by supercapacitors (SCs) and lithium-ion batteries (LIBs) have gathered global concern and extensive investigation. The former has overwhelming advantages on power density and lifespan, but is limited by low energy density (Wang et al., 2017a; Guan et al., 2020). The later has been employed as the predominant power source for portable electronics due to their high energy density (Teng et al., 2020). In particular, silicon, as the second-most abundant element on the Earth, can offer ultra-high theoretical capacity of 4,000 mA h g^−1^, which is more than ten times that of traditional graphite anode, so high-energy-density LIBs using silicon-based anodes show a vigorous development momentum (Jin et al., 2017; Zuo et al., 2017). Unfortunately, they suffer from insufficient power density and short lifespan. In the perspective of the burgeoning development of electric vehicles and grid energy-storage systems, it is urgently desirable for the cutting-edge energy storage systems combining with high energy density and high power output. The recently emerging lithium-ion capacitors (LICs), which are constructed with capacitor-type cathodes and battery-type anodes, are considered to be one of the most promising candidates to bridge the gap between SCs and LIBs (Ding et al., 2018). Capacitor-type cathodes can afford high power density because their charge storage hinges on the fast and reversible adsorption/desorption of ions on electrode surface (Li et al., 2018; Jagadale et al., 2019). For the battery-type anodes, the capacity originates from the intercalation/deintercalation of lithium ions into/from the bulk of electrode. In spite of high energy density, the power density sacrifices because the sluggish diffusion kinetics in the bulk does not match the fast electrostatic accumulation behavior of cathodes, and the lithium storage process is usually accompanied by the destruction of the electrode structure, which will result in fast capacity fading (Aravindan et al., 2014; Han et al., 2018b). Therefore, it is of great significance to explore novel anode materials with fast reaction kinetics and well-designed microstructure towards the further improvement of LICs.

Carbon materials are regarded as the ideal anode materials for LICs due to their excellent electrical conductivity, stable electrochemical properties and easy preparation (Han et al., 2018a; Wang et al., 2019b). The nanostructure engineering and composition optimization of carbon materials have been extensively conducted to improve the electrochemical performances of carbon anodes. Benefiting from the high surface-to-volume ratio, hollow nanostructure can provide more accessible storage sites for Li ions and large contact interface between electrode and electrolyte, thus rendering increased capacity and shortened ion/electron-transport path. Besides, the interior cavity of such a nanostructure can also accommodate the volume expansion and alleviate the stress/strain during repetitive lithiation/delithiation process (Wang et al., 2019a; Liu et al., 2019b). The doping of heteroatoms [such as N (Peng et al., 2020; Zou et al., 2020; Zou et al., 2021), O (Liu et al., 2019a; Wang et al., 2021), B (Xia et al., 2017; Lee and An, 2020) and P (Luan et al., 2019; Hu et al., 2020) etc.] is another feasible route to propel the reaction kinetics of carbon anodes by introducing additional active sites for rapid pseudocapacitive reaction.

Conductive polymers with π-conjugated system have been demonstrated to promote the electrochemical performances of original electrodes in various energy storage fields by virtue of their appealing properties including high electrical conductivity and reasonable mechanical resilience (Shi et al., 2015; Nnadiekwe et al., 2021). More interestingly, it was recently reported that polypyrrole (PPy), a representative conductive polymer, can undergo reversible redox reactions with lithium ions when used as the anode of LIBs, indicating a considerable capacitance contribution (Numazawa et al., 2018). Therefore, PPy holds a great application potential in anodes for high-performance LICs, which needs to be further explored.

Taking all these considerations into account, we propose a strategy of template-assisted carbonization followed by polymerization to synthesize PPy-coated nitrogen and phosphorus co-doped hollow carbon nanospheres (NPHCS@PPy) and investigate the electrochemical performances of the composite as the anode material for LIC device. In the hierarchical architecture, the carbon nanospheres with porous hollow nanostructure as well as ample N and P heteroatoms can facilitate the high-efficiency lithium storage, and the PPy coating layer not only functions as the conductive and flexible network between carbon nanospheres, but also contributes to additional capacity. Thus, the LIC device assembled with NPHCS@PPy as the anode and nitrogen-doped porous carbon (NPC) as the cathode achieves high energy density and power density as well as long cycle lifespan.

## Experimental Section

### Synthesis of Nitrogen/Phosphorous Co-Doped Hollow Carbon Nanospheres

24 mmol of tetraethyl orthosilicate (TEOS) and 6 ml of NH_3_·H_2_O were added into the mixed solution of deionized water and ethanol (7:1 by volume) and stirred for 30 min to form a milky white suspension. Subsequently, 0.8 g of resorcinol, 1.12 ml of formaldehyde and 3 g of ammonium phosphate monobasic were dipped into the above mixture followed by stirring for 24 h. The powder was collected by filtering, washing with deionized water and ethanol, and drying at 80°C for 24 h, and then was thermally treated at 900°C for 4 h under N_2_ gas flow with a heating rate of 5°C min^−1^. Finally, SiO_2_ templates were removed by washing with 2 mol L^−1^ of NaOH solution for 12 h, and nitrogen and phosphorus co-doped hollow carbon nanospheres, denoted as NPHCS, were obtained after filtering and drying. For comparison, hollow carbon nanospheres (HCS) were fabricated without adding ammonium phosphate monobasic.

### Synthesis of NPHCS@PPy

100 mg of NPHCS was dispersed in FeCl_3_ aqueous solution (3.84 mM, 100 ml), and then 45 μL of pyrrole was dropwise added into the above suspension within 10 min and kept stirring for 24 h under ice bath. Finally, the obtained NPHCS@PPy composite was thoroughly washed with deionized water *via* filtration and dried at 80°C for 12 h under vacuum. For comparison, HCS@PPy was fabricated under the same conditions.

### Synthesis of NPC

The mixture of citric acid trisodium and urea with a mass ratio of 1:1 was sufficiently grinded and then carbonized at 600°C for 2 h under N_2_ gas flow with a heating rate of 2°C min^−1^. The product was soaked in 0.1 mol L^−1^ of HCl solution and washed with deionized water for several times. After drying, the as-obtained sample and KOH powder were mixed with a mass ratio of 1:1 and then activated at 700°C for 2 h under N_2_ gas flow with a heating rate of 5°C min^−1^. Finally, residual KOH was removed by washing with 0.1 mol L^−1^ of HCl solution, and NPC was obtained after filtering and drying.

### Material Characterization

Field emission scanning electron microscope (FESEM) observation was carried out on Hitachi S4800. Transmission electron microscopy (TEM) images with EDS element mapping were obtained on JEM-2010 system. The phase structure and composition were detected by X-ray diffraction (XRD, X'Pert PRO MPD diffractometer), Raman spectroscopy (Renishaw RM 2000) and Fourier transform infrared (FT-IR) absorption spectroscopy (NEXUS FT-IR). The Brunauer-Emmet-Teller (BET) specific surface area and pore characteristics were measured by N_2_ physical adsorption instrument (Micromeritics ASAP 2020 analyzer). The surface chemical status was decided by X-ray photoelectron spectroscopy (XPS, Thermo Scientific Escalab 250XI).

### Electrochemical Measurement

The NPHCS@PPy anode and NPC cathode were prepared by coating slurries on copper foil and aluminum foil, respectively. The slurries were composed of active materials (80 wt%), super P (10 wt%) and polyvinylidene fluoride (PVDF, 10 wt%) in the N-methy-2-pyrrolidone (NMP) dispersant. Subsequently, the electrodes were dried at 80°C for 12 h under vacuum to remove NMP and then were cut into film disks. The mass loading of the NPHCS@PPy anode and NPC cathode was about 0.8 and 1.2 mg cm^−2^, respectively.

The electrochemical performances of the anode and cathode were separately evaluated in a half cell, in which the as-made anode or cathode was used as the working electrode, lithium foil was concurrently used as the counter electrode and reference electrode, polypropylene film (Celgard 2,400) was applied as the separator, and 1 M LiPF_6_ in dimethyl carbonate and ethylene carbonate (1:1 by volume) was employed as the electrolyte. The potential range of the anode and cathode was 0.01–3 V and 2–4.5 V vs. Li/Li^+^, respectively. The LIC device was constructed using the NPC cathode and the pre-lithiated NPHCS@PPy anode which was prepared by discharging and charging in the half cell for five cycles at a current density of 0.1 A g^−1^ and finally discharging to 0.01 V. The mass ratio of cathode and anode was 1.5:1. A reasonable voltage window of the LIC device was selected to be 0.5–4 V using a three-electrode system with the addition of lithium reference electrode. All cells were assembled in an Ar-filled glovebox (H_2_O < 0.1 ppm, O_2_ < 0.1 ppm). The cyclic voltammetry (CV) measurement and electrochemical impedance spectroscopy (EIS, 100 kHz to 10 mHz) were performed on CHI760D electrochemical working station, and the galvanostatic charge-discharge (GCD) tests were carried out on LAND CT 2001A test system. The energy density (*E*, Wh kg^−1^) and power density (*P*, W kg^−1^) of the LIC device were analyzed based on the following equations (Li et al., 2021):

$$P=\frac{I\times \Delta V}{m}$$ (1)

E=P×t (2)

$$\Delta V=\frac{V_{max}+V_{min}}{2}$$ (3)

Where *I* is the discharge current (A), *t* is the discharge time (s), *m* is the total mass of active materials on anode and cathode (kg), and *V*_max_ and *V*_min_ are the voltage at the beginning and the end of discharge process (V), respectively.

## Results and Discussion

### Microstructure and Composition Analysis of NPHCS@PPy Anode

**FIGURE 1:**
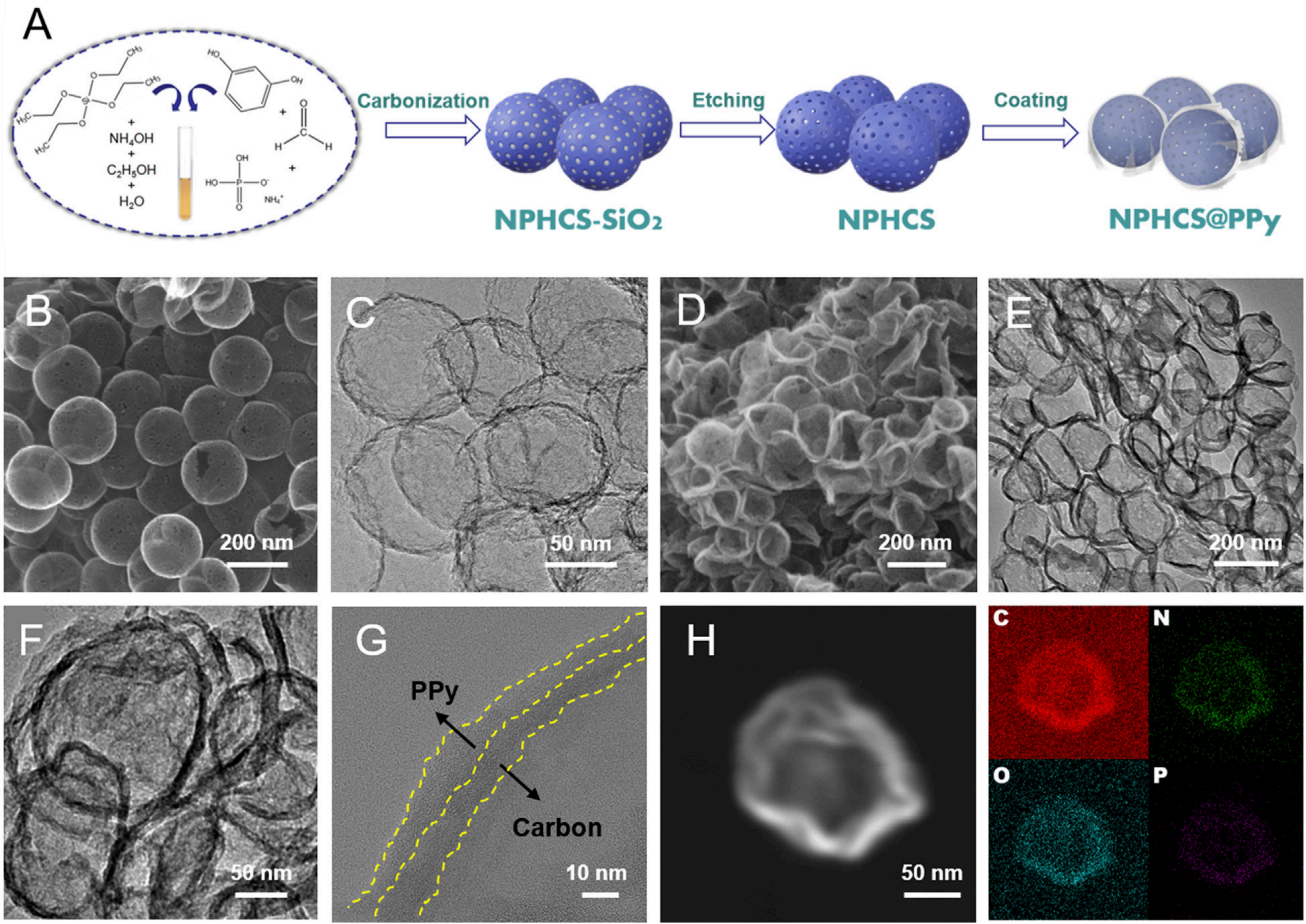
**(A)** Synthesis schematic of NPHCS@PPy **(B)** SEM and **(C)** TEM images of NPHCS **(D)** SEM **(E, F)** TEM **(G)** HR-TEM and **(H)** EDS elemental mapping images of NPHCS@PPy.

The synthetic process of NPHCS@PPy composite is illustrated in Figure 1A. NPHCS was first synthesized by using silica as hard templates, a polymer of resorcinol and formaldehyde as the carbon source, and ammonium dihydrogen phosphate as the nitrogen and phosphorous source. The SEM images of carbonized products (NPHCS-SiO_2_) shown in Supplementary Figure S1 demonstrate the uniform spherical morphology with a diameter size of 180 nm. After the removal of silica templates, the spherical structure is preserved and a wealth of mesopores are observed on the surface of spheres (Figure 1B). The TEM image (Figure 1C) further reveals the hollow nanostructure of NPHCS. Afterwards, conductive PPy was coated on the surface of NPHCS *via* an *in-situ* polymerization reaction. As we can see from the SEM and TEM images of NPHCS@PPy (Figures 1D–F), a multitude of deformed hollow nanospheres are adhered to each other, and the shell thickness significantly increases, indicative of the successful cladding of PPy. The HR-TEM image (Figure 1G) further reveals the double-shell structure, and the thickness of both inner carbon layer and outer PPy layer is a few nanometers. Moreover, EDS elemental mapping images (Figure 1H) of NPHCS@PPy composite show that C, O, N, and P elements are uniform distributed in the shell of nanospheres, indicative of the successful dual doping of N and P atoms.

**FIGURE 2:**
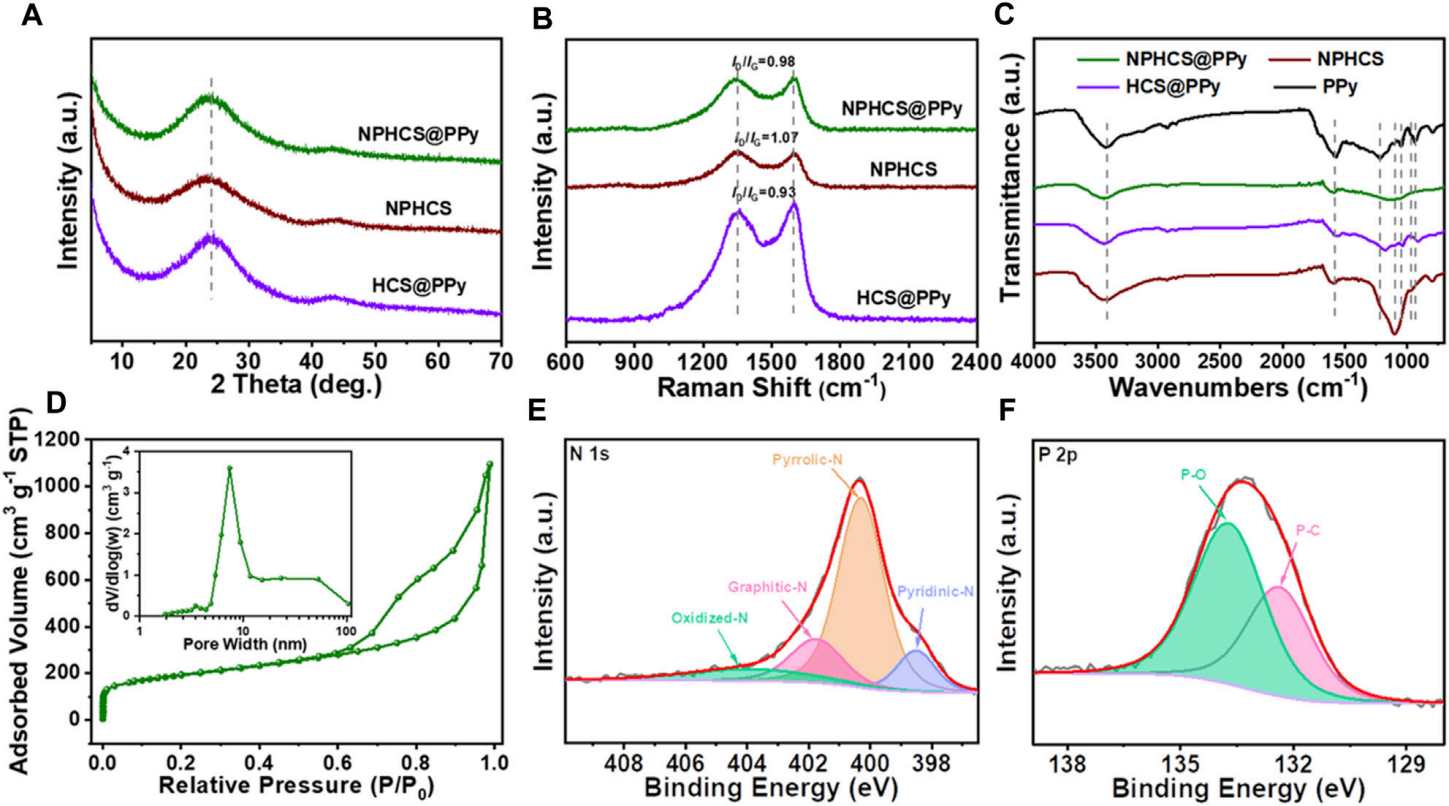
**(A)** XRD patterns and **(B)** Raman spectra of NPHCS@PPy, NPHCS, and HCS@PPy **(C)** FT-IR spectra of pure PPy, NPHCS@PPy, NPHCS, and HCS@PPy **(D)** N_2_ adsorption and desorption isotherms of NPHCS@PPy with the inset of their pore size distribution curves. High-resolution XPS spectrum of **(E)** N1s and **(F)** P2p for NPHCS@PPy.

To gain further insights into the structure and composition of NPHCS@PPy, the characterizations including XRD, Raman, FT-IR, nitrogen adsorption/desorption experiment and XPS have been carried out. As shown in the XRD patterns (Figure 2A), NPHCS@PPy exhibits two diffraction peaks at 23.6° and 43.1°, which are assigned to (002) and (100) crystal planes of graphite, respectively. Moreover, compared to HCS@PPy, the (002) peaks of both NPHCS and NPHCS@PPy samples become weaker and shift to a lower diffraction angel, demonstrating that the disorder degree and interlayer distance increase after N and P atoms are doped in the carbon lattice. In addition, there are no characteristic peaks of PPy in NPHCS@PPy and HCS@PPy samples, indicative of the amorphous structure of PPy coating layer (Wang et al., 2016a; Luo et al., 2019). As shown in Figure 2B, there are two peaks at 1,348 and 1,596 cm^−1^ in the Raman spectra of all samples, corresponding to the disorder-related D band and sp^2^ hybridized G band, respectively. The higher *I*_D_/*I*_G_ values of NPHCS and NPHCS@PPy than HCS@PPy also demonstrate more structure defects introduced by N and P co-doping, which aid in improving the accessibility of Li ions to the active sites (Niu et al., 2018). As we can see from Figure 2C, the FT-IR spectrum of NPHCS displays the main bands centered at 3,425, 1,586, 1,102, and 780 cm^−1^, which are assigned to N-H, C = C, O-H, and C-H bond, respectively. In the case of NPHCS@PPy and HCS@PPy, the intensity of above peaks is obviously weaker, and new characteristic peaks appear at the region of 1,200–900 cm^−1^, further confirming the successful coating of PPy. In addition, slight peak position shifts can be observed, which may suggest the mutual interaction between hollow carbon nanospheres and PPy coating layer (Hu et al., 2015; Han et al., 2019). As shown in Figure 2D, the nitrogen adsorption and desorption isotherms of NPHCS@PPy composite present a representative type II isotherms with a H3-type hysteresis loop at the relative pressure over 0.6, indicative of the hierarchical porous structure. The pore distribution curve depicted in the insert further verifies the combination of mesopores and macropores. The BET specific surface area and pore volume of NPHCS@PPy are 649 m^2^ g^−1^ and 1.72 cm^3^ g^−1^, respectively. The full XPS spectra (Supplementary Figure S2A) reveal the successful doping of N and P elements in the NPHCS (3.1 at%, 1.5 at%) and NPHCS@PPy (6.5 at%, 2.4 at%), and the N content of the latter dramatically increases owing to the surface coating of N-rich polymers. The high-resolution N1s XPS spectra (Figure 2E and Supplementary Figures S2B,C) indicate that there are four types of doped N including pyridinic N, pyrrolic N, graphitic N, and oxidized N in three samples. As revealed by Supplementary Figure S3, the dominant N specie in the NPHCS sample is graphitic N which is conductive to facilitating electron transport *via* carbon plane (Wang et al., 2021), and the proportion of pyrrolic N specie is the highest in the NPHCS@PPy sample, further manifesting the presence of PPy. Two fitted peaks in the P 2p XPS spectrum (Figure 2F) are assigned to P-C bond (132.4 eV) and P-O bond (133.7 eV), respectively. These abundant N and P heteroatoms can induce surface polarization of carbon matrix, which is favorable for improving the electrode wettability and creating more active sites for enhanced ion attraction (Hu et al., 2020).

### Electrochemical Performances of NPHCS@PPy Anode

**FIGURE 3:**
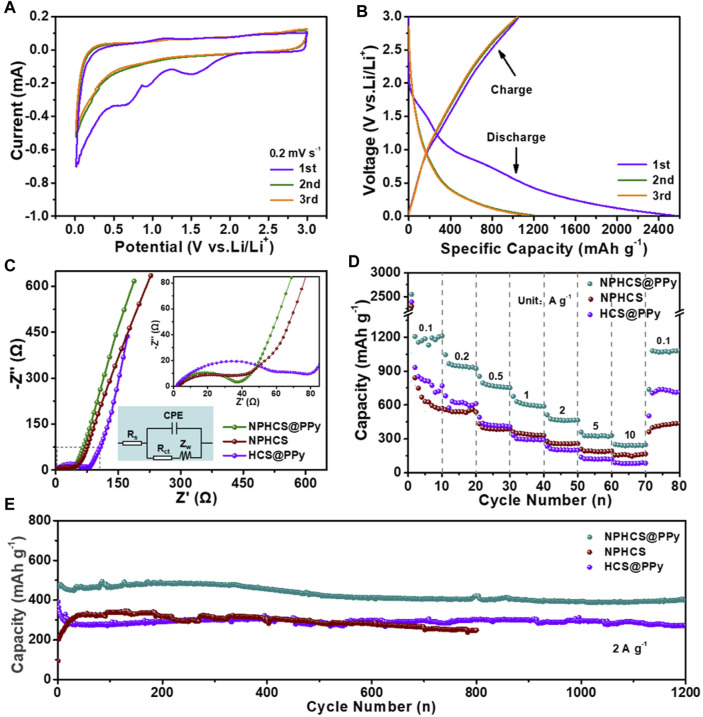
**(A)** CV curves of NPHCS@PPy anode for the first three cycles at a scan rate of 0.2 mV s^−1^ **(B)** GCD profiles of NPHCS@PPy anode for the first three cycles at a current density of 0.1 A g^−1^ **(C)** Nyquist plots with the insert of equivalent circuit model **(D)** rate performances and **(E)** cycling performances of NPHCS@PPy, NPHCS, and HCS@PPy.

The lithium storage capability of as-made anode materials was evaluated in a half cell configuration. Figure 3A and Supplementary Figures S4A,B present the CV curves for initial three cycles at a scan rate of 0.2 mV s^−1^ in the potential range of 0.01–3 V vs. Li/Li^+^. In the first cycle, two board reduction peaks can be observed below 1.2 V for all anode materials, which can be attributed to the decomposition of electrolyte and the generation of solid electrolyte interface (SEI) film (Zheng et al., 2014; Zou et al., 2021). Moreover, the peak intensity of NPHCS@PPy and NPHCS is obviously weaker than that of HCS@PPy, which demonstrates the doping of N and P heteroatoms could refrain the decomposition of electrolyte and side reaction between electrode surface and electrolyte to form SEI film (Wu et al., 2011). Another reduction peak at around 1.53 V is probably ascribed to the irreversible reactions between surface functional group and electrolyte (Xia et al., 2017). In the subsequent cycles, these reduction peaks disappear and the CV curves are almost overlapping, indicative of the good stability and reversibility. A slope profile can be observed from GCD profiles of three anode materials shown in Figure 3B and Supplementary Figures S4C,D. And the NPHCS@PPy anode exhibit the highest discharge capacity (2,549 mA h g^−1^) and charge capacity (1,056 mA h g^−1^) with the highest initial Coulombic efficiency of 41.4%. Figure 3C shows the electrochemical impedance spectroscopy (EIS) of three fresh cells. The NPHCS@PPy anode exhibits the minimum charge transfer resistance (*R*_ct_), indicating the fast reaction kinetics on the surface of electrode. This could be attributed to the outstanding electrical conductivity combined with high-efficient faradaic redox reactions provided by PPy coating layer and co-doped N/P heteroatoms. The comparison of rate performances of three anode materials is displayed in Figure 3D. The NPHCS@PPy anode possesses the highest capacity at the same current density. Even when the current density is as high as 5 and 10 A g^−1^, the specific capacity of NPHCS@PPy anode still maintains 329 and 250 mA h g^−1^, respectively. Also, most of the capacity can recover when the current density is back to 0.1 A g^−1^, indicative of the high electrochemical reversibility. As shown in Figure 3E, the NPHCS@PPy anode achieves the best cycling performance. A high capacity of 400 mA h g^−1^ is retained after 1,200 cycles at a high current density of 2 A g^−1^. In addition, NPHCS@PPy possesses a larger electrochemically active surface area (ECSA) than HCS@PPy, further demonstrating that dual-doped N and P heteroatoms can provide more active sites for fast faradaic redox reactions (Supplementary Figures S5A–C).

The capacity storage mechanism of NPHCS@PPy was further investigated. Generally, the relationship between current (i) and scan rate (v) obeys the following equation (Wang et al., 2017b):

$$i=av^{b}$$ (4)

where a and b stand for adjustable constants. b = 0.5 indicates a semi-infinite diffusion-controlled process, and b = 1 represents a surface-dominated process. As shown in Supplementary Figure S5D, the b value is calculated to be 0.69 by linearly fitting the log(*i*)-log(*ν*) points, indicating a tendency of capacitive kinetics. According to the Dunn’s method, the current at a fixed potential can also be described as the following equation (Wang et al., 2007):

$$i=k_{1}v+k_{2}v^{1/2}$$ (5)

$${i/v}^{1/2}=k_{1}v^{1/2}+k_{2}$$ (6)

where k_1_ *ν* refers to a capacitive contribution and k_2_ *ν* ^1/2^ refers to a diffusion-controlled contribution. The constant k_1_ and k_2_ can be determined by plotting the *i*/ν^1/2^ versus ν^1/2^ at different potentials. By comparing the shaded area (k_1_ *v*) with the experimental currents in Supplementary Figure S5E, approximately 62.5% of total capacity is derived from the capacitive contribution at a scan rate of 1 mV s^−1^. Correspondingly, the capacity contribution at different scan rates are also determined and shown in Supplementary Figure S5F. It is found that the capacitive contribution increases with the increase of scan rates owing to highly efficient pseudocapacitive reaction induced by abundant N and P heteroatoms at high rates. All above electrochemical results elucidate that the NPHCS@PPy anode possesses the best lithium-storage capability, which can be attributed to the comprehensive advantages from the carbon spheres with hollow porous structure and abundant heteroatom doping and the PPy coating layer with high conductivity and flexibility.

### Microstructure and Composition Analysis of NPC Cathode

The key to constructing high-energy LICs is the preparation of cathode materials with high capacity. In this work, the NPC cathode material was synthesized by a carbonization assisted activation method. The SEM images (Supplementary Figures S6A,B) reveal its sheet-like structure with interconnected macropores, and the TEM images (Supplementary Figures S6C,D) further indicate that there are a multitude of micropores and mesopores on the surface of nanosheets. The well-developed pore structure was also demonstrated by the nitrogen adsorption experiment (Supplementary Figures S7A,B). NPC has a BET specific surface area up to 2,307 m^2^ g^−1^, and the pore size distribution is mainly centered at the range of 1.7–5.0 nm. Moreover, the NPC sample presents a weak (002) diffraction peak in the XRD pattern (Supplementary Figure S7C) and a high *I*_D_/*I*_G_ value in the Raman spectrum (Supplementary Figure S7D), which means a large number of defects in the carbon lattice caused by abundant in-plane pores and doped N heteroatoms. The full XPS spectrum of NPC (Supplementary Figure S7E) demonstrates the successful doping of N with a high content of 6.5 at.%, and the N species can be divided to pyridinic N (398.5 eV), pyrrolic N (399.9 eV), graphitic N (400.7 eV), and oxidized N (402.5 eV) based on the fitting results of high-resolution N1s XPS spectrum (Supplementary Figure S7F). These intriguing features including large specific surface area and high N doped level endow NPC with a great promise as the cathode material for high-performance LICs.

### Electrochemical Performances of NPC Cathode

The electrochemical performances of NPC cathode were evaluated in a half cell with Li foil as both anode and reference electrodes. A quasi rectangle with humps can be observed from all CV curves (Supplementary Figure S8A) at different scan rates, which indicates the dominating electrochemical double-layer capacitance (EDLC) derived from the adsorption/desorption of PF_6_^−^ ions and subordinate pseudocapacitance derived from the Faradaic reaction between electrolyte and heteroatoms. The GCD profiles (Supplementary Figure S8B) present nearly symmetric triangle shape without severe deviation even at high current density, further confirming a good EDLC behavior, and good electrochemical reversibility. As shown in Supplementary Figure S8C, the NPC cathode exhibits excellent rate performance with a high capacity of 86 mA h g^−1^ even at a high current density of 10 A g^−1^. Also, an ultralong lifespan over 10,000 cycles can be achieved at a current density of 2 A g^−1^ (Supplementary Figure S8D). The EIS curves of NPC cathode before cycle and after 1,000 cycles are shown in Supplementary Figure S8E. With the cycle proceeding, the *R*_ct_ obviously decreases due to the electrochemical activation of electrode, and such a small *R*_ct_ value of 4 *Ω* suggests the fast reaction kinetics on the electrode surface. These electrochemical results demonstrate that NPC is more suitable than active carbon as the cathode material for high-energy LICs (Wang et al., 2016b; Shi et al., 2018).

### Electrochemical Performances of NPHCS@PPy//NPC LIC Device

**FIGURE 4:**
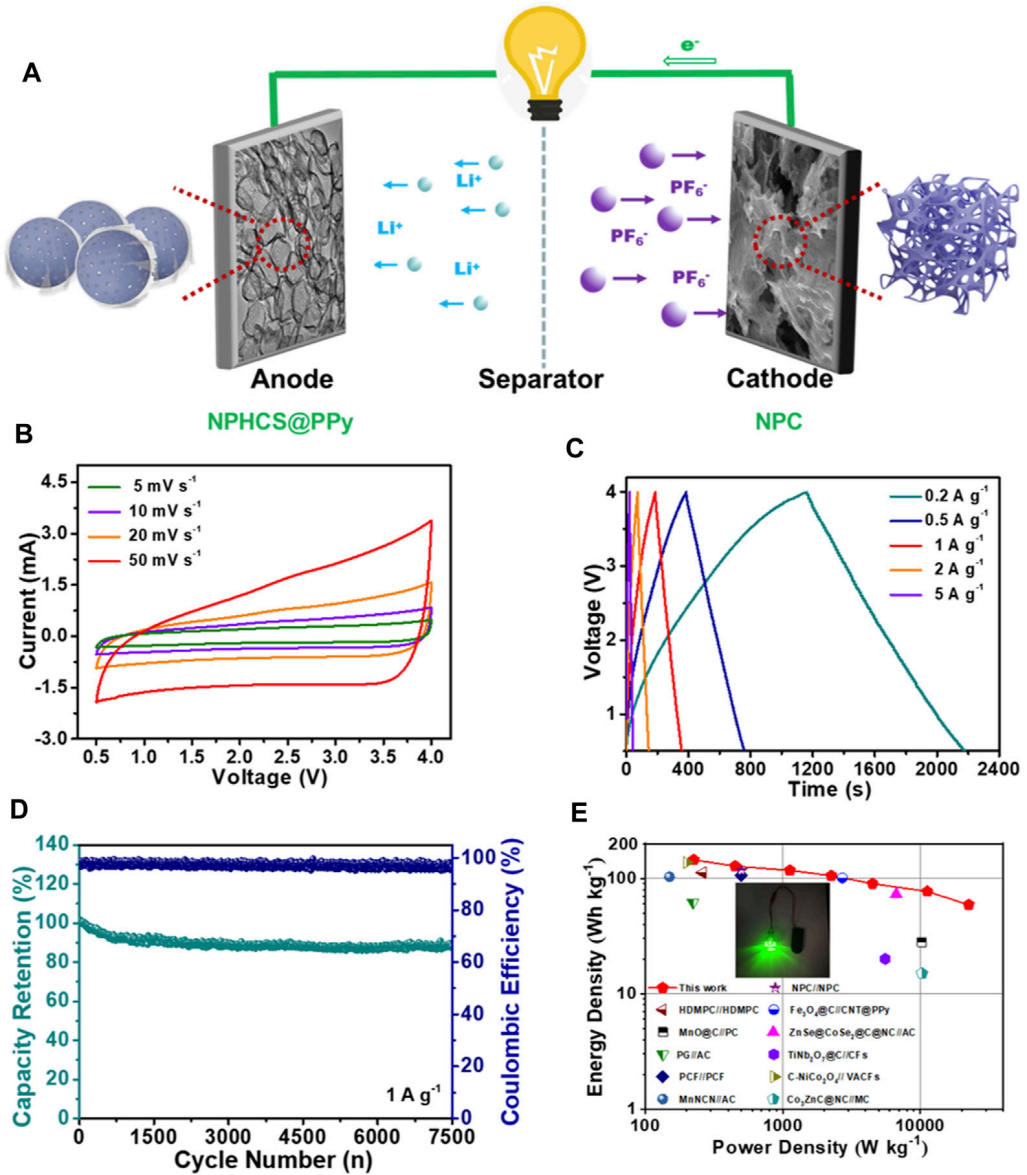
Electrochemical performances of NPHCS@PPy//NPC LIC **(A)** Schematic illustration of the charge process **(B)** CV curves at various scan rates **(C)** GCD profiles at various current density **(D)** Cycling performance at a current density of 1 A g^−1^ **(E)** Ragone plots in comparison with other reported LICs.

In view of good lithium storage performance of NPHCS@PPy and excellent charge storage capability of NPC, a full LIC device (denoted as NPHCS@PPy//NPC) was assembled using prelithiated NPHCS@PPy as the anode and NPC as the cathode, as schematically shown in Figure 4A. During the charge process, PF_6_^−^ ions are adsorbed on the porous surface and defects moieties of NPC cathode, while Li^+^ ions are intercalated into the interlayer or adsorbed on the surface of NPHCS@PPy anode. A reverse behavior occurs during the discharge process. The LIC device can work in a broad and safe voltage window of 0.5–4 V without electrolyte decomposition and cathodic reaction (Supplementary Figure S9) (Cao and Zheng, 2013; Dubal et al., 2019). It can be seen from Figure 4B that the quasi rectangular shape of CV curves can be well retained with the increase of scan rates, indicative of good capacitive behavior and good rate capability. The GCD files (Figure 4C) show near-liner slope at various current density, revealing outstanding electrochemical reversibility. More remarkably, the LIC device achieves a marvelous cycling performance with a capacity retention of up to 92% after 7,500 cycles at a current density of 1 A g^−1^ (Figure 4D). The change of EIS curves before and after 1,000 cycles manifests the electrode kinetics is significantly accelerated after an electrochemical activation process (Supplementary Figure S10). The energy density and power density of the NPHCS@PPy//NPC LIC were calculated based on the GCD profiles and depicted in the Ragone plot (Figure 4E). The device achieves a high energy density of 145 W h kg^−1^ at a power density of 225 W kg^−1^ and still retains a decent energy density of 59 W h kg^−1^ even at a high power density of 22,500 W kg^−1^, which are superior to previously reported LICs, such as N-doped porous carbon (NPC)//NPC (Zou et al., 2020), High-defect mesopore-dominant porous carbon (HDMPC)//HDMPC (Niu et al., 2018), Fe_3_O_4_@C//CNT@PPy (Han et al., 2019), MnO@C//Porous carbon (PC) (Yan et al., 2018), ZnSe@CoSe_2_@C@N-doped carbon (NC)//AC (Chen et al., 2020), Pre-lithiated graphene (PG)//AC (Ren et al., 2014), TiNb_2_O_7_@C//Carbon fibers (CFs) (Wang and Shen, 2015), Porous carbon frameworks (PCF)//PCF (Qian et al., 2021), Carbonized NiCo_2_O_4_ (C-NiCo_2_O_4_)//Vertically aligned carbon nanoflakes (VACFs) (Cheng et al., 2019), MnNCN//AC (Liu et al., 2017) and Co_3_ZnC@N-doped carbon (NC)//Mesoporous carbon (MC) (Zhu et al., 2018). The detail comparison are shown in Supplementary Table S1. Furthermore, the potential applications of NPHCS@PPy//NPC LIC could be demonstrated by a light-emitting diode powered by this device (Figure 4E, inset).

## Conclusion

In summary, we present a strategy of template-assisted carbonization followed by polymerization to the synthesis of NPHCS@PPy composite featuring a large specific surface area, hierarchically porous structure, enlarged interlayer space, abundant N and P heteroatoms as well as highly conductive and flexible PPy coating. Benefiting from the unique structure and composition advantages, the NPHCS@PPy composite possesses fast and robust lithium storage properties dominated by the surface pseudocapacitive behavior. In addition, the as-made NPC material with well-developed pore structure and abundant N heteroatoms exhibits outstanding charge storage capability. Consequently, the NPHCS@PPy//NPC LIC device achieves impressive electrochemical performances including high energy density (149 Wh kg^−1^), high power density (22,500 W kg^−1^) and superior cycling stability (92% capacity retention after 7,500 cycles), which demonstrate the glorious application prospects of NPHCS@PPy as the anode material for high-performance LICs.

## Data Availability

The original contributions presented in the study are included in the article/Supplementary Material, further inquiries can be directed to the corresponding authors.
