# Supplementary material for: Robust and Fast Lithium Storage Enabled by Polypyrrole-Coated Nitrogen and Phosphorus Co-Doped Hollow Carbon Nanospheres for Lithium-Ion Capacitors
Source: Front Chem. 2021 Sep 24;9:760473. doi: 10.3389/fchem.2021.760473 (PMC8497749; doi:10.3389/fchem.2021.760473)
Supplement: Supplementary file 1 [file DataSheet1.docx]

Supplementary Material

Robust and Fast Lithium Storage Enabled by Polypyrrole-Coated Nitrogen and Phosphorus Co-Doped Hollow Carbon Nanospheres for Lithium-Ion Capacitors

Mengdi Zhang^1^, Xuan Zheng^1^, Jiawei Mu^1^, Pengfei Liu^1^, Wenhan Yuan^1^, Shuli Li^1^, Xiaobo Wang^1^, Haiqiu Fang^1^, Haiyan Liu^2^, Tao Xing^2^, Han Hu^1*^, Mingbo Wu^1*^

^1^State key laboratory of Heavy Oil Processing, Institute of New Energy, College of Chemical Engineering, China University of Petroleum (East China), Qingdao, 266580, China

^2^New Energy Division, ShanDong Energy Group CO., LTD., Zoucheng 273500, China

*** Correspondence:**Han Hu, hhu@upc.edu.cn; Mingbo Wu, wumb@upc.edu.cn


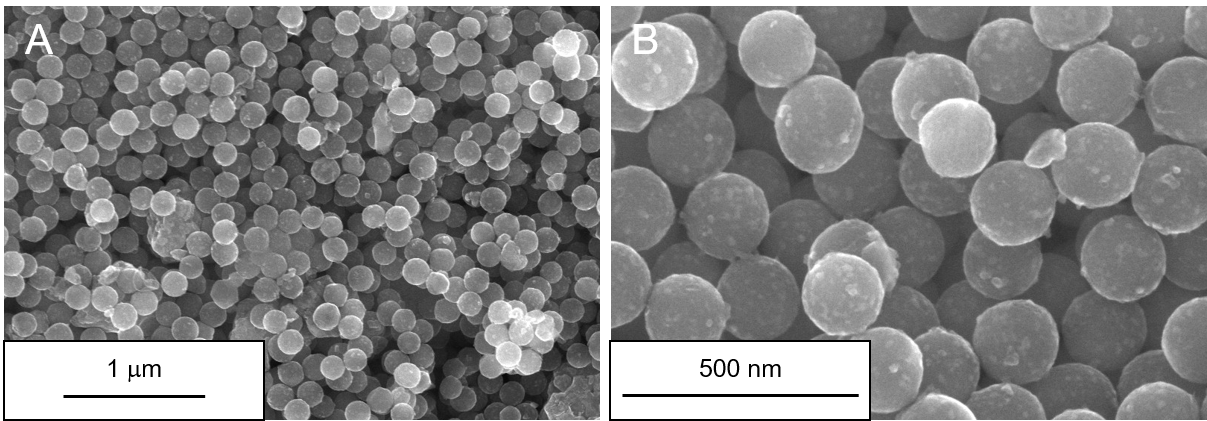


**FIGURE S1.** SEM images of NPHCS-SiO_2_.


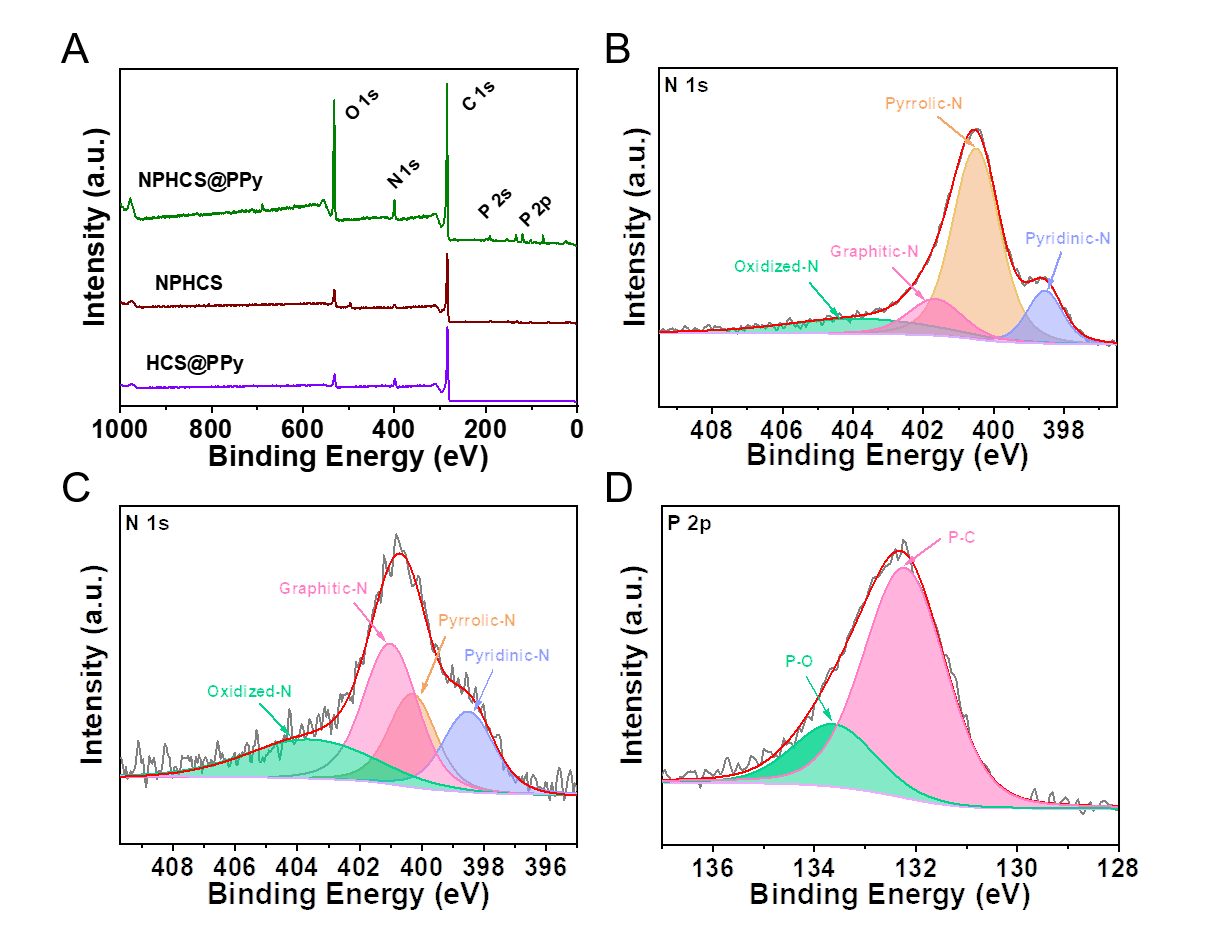


**FIGURE S2.** **(A)** Full XPS spectra of NPHCS@PPy, NPHCS and HCS@PPy. **(B)** High-resolution N 1s XPS spectrum of HCS@PPy. High-resolution XPS spectra of **(C)** N1s and **(D)** P2p for NPHCS.


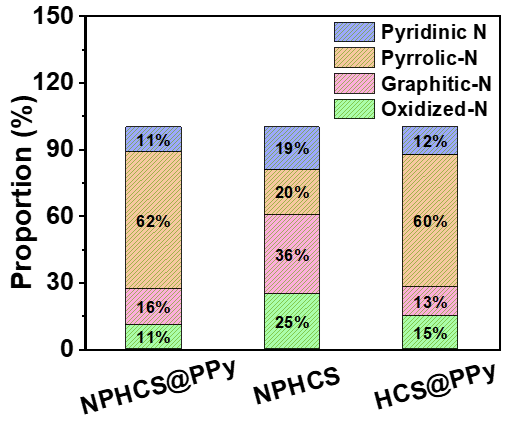


**FIGURE S3.** Proportion of various nitrogen species in NPHCS@PPy, NPHCS and HCS@PPy.


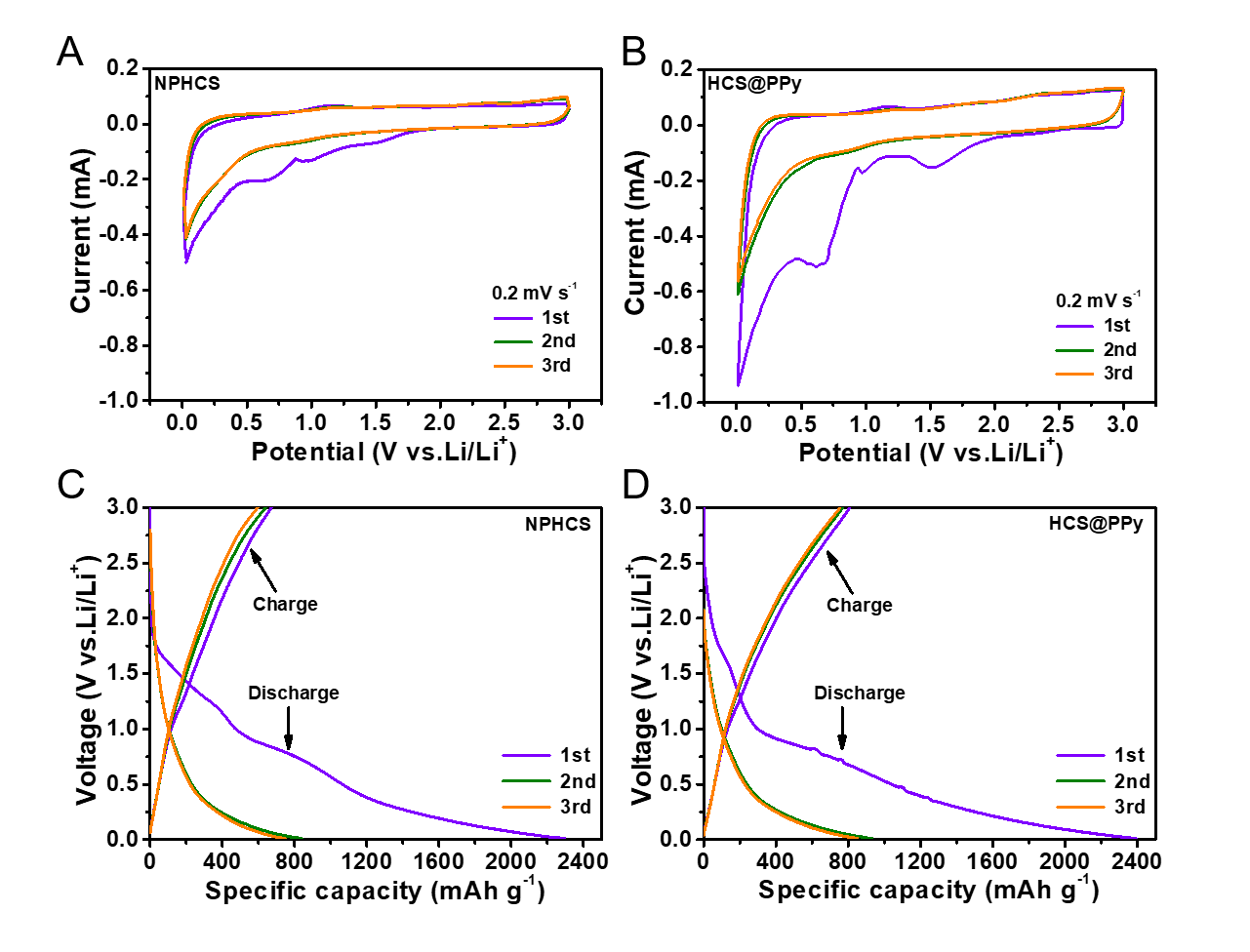


**FIGURE S4.** CV curves of **(A)** NPHCS and **(B)** HCS@PPy anodes for the first three cycles at a scan rate of 0.2 mV s^-1^. Galvanostatic charge-discharge profiles of **(C)** NPHCS and **(D)** HCS@PPy anodes for the first three cycles at a current density of 0.1 A g^-1^.


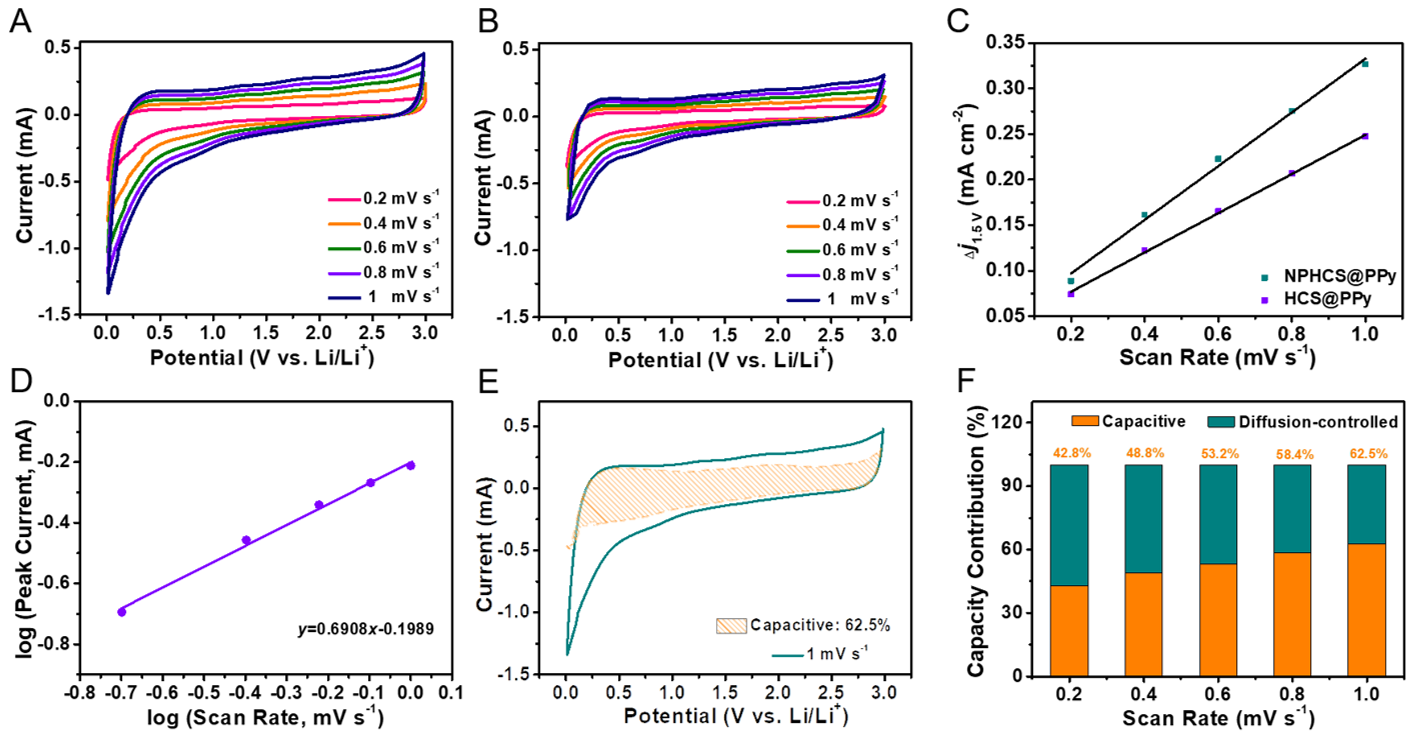


**FIGURE S5.** CV curves of **(A)** NPHCS@PPy and **(B)** HCS@PPy anodes at various scan rates. **(C)** The difference between the anodic and cathodic current density (△*j*=*j*_a_-*j*_c_) versus scan rate. Capacity storage mechanism analysis for NPHCS@PPy anode: **(D)** The power law relationship between peak current and scan rate, **(E)** Capacitance contribution obtained from the CV curve of 1 mV s^-1^, **(F)** Capacity contribution proportions at different scan rates.

The differences in electrochemically active surface area (ECSA) could be estimated from the electrochemical double-layer capacitance (EDLC, *C*_dl_) property (Pu et al., 2019;Qin et al., 2020;Liu et al., 2021). As shown in Figures S5A,B, the CV measurements of NPHCS@PPy and HCS@PPy were carried out at various scan rates from 0.2 to 1 mV s^-1^. The difference between the anodic and cathodic current density at 1.5 V was then plotted as a function of the scan rate. As show in Figures S5C, the slopes of the fitting lines could be used to determine *C*_dl_, and the large slope represents the large ECSA.


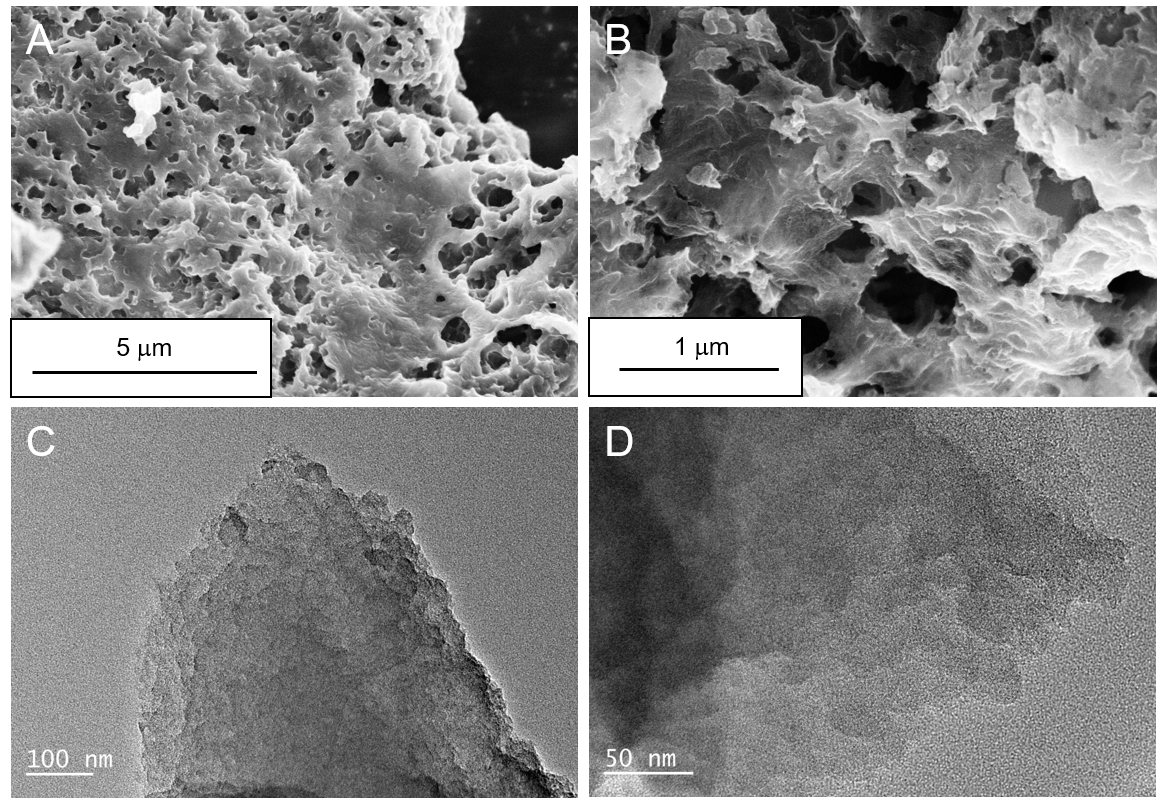


**FIGURE S6.** **(A, B)** SEM and **(C, D)** TEM images of NPC.


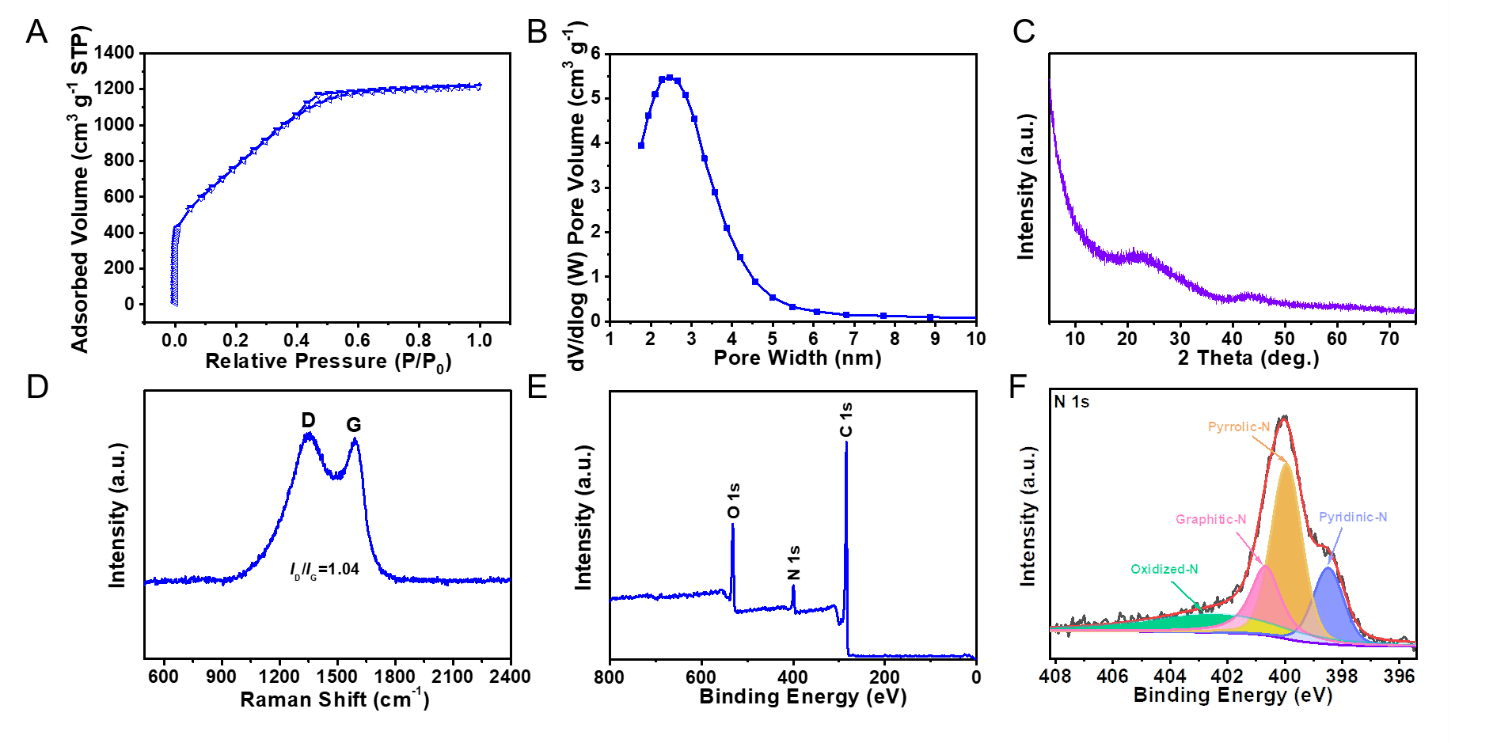


**FIGURE S7.** Structure and composition characterization of NPC. **(A)** N_2_ adsorption and desorption isotherms. **(B)** Pore size distribution. **(C)** XRD pattern. **(D)** Raman spectrum. **(E)** Full XPS spectrum. **(F)** High-resolution N1s XPS spectrum.


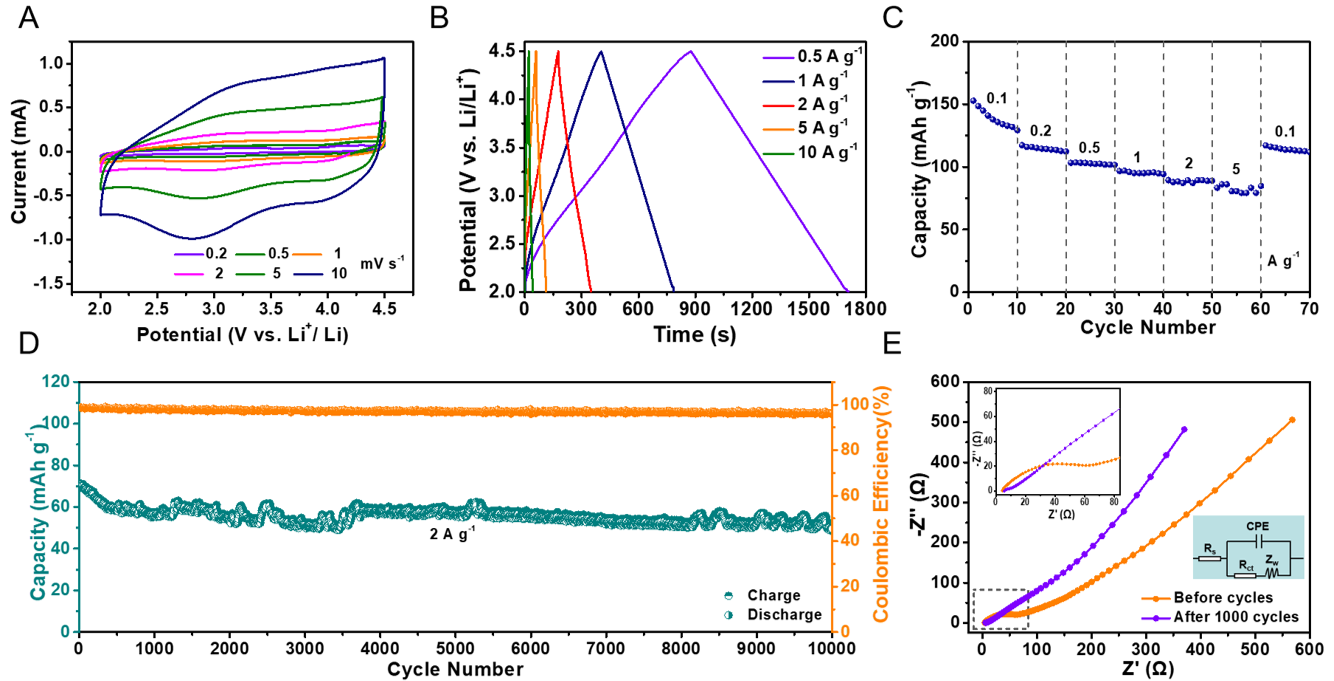
**FIGURE S8.** Electrochemical performances of NPC cathode in a half cell. **(A)** CV curves at various scan rates. **(B)** Galvanostatic charge-discharge profiles at various current density. **(C)** Rate performance. **(D)** Cycling performance at a current density of 2 A g^-1^. **(E)** Nyquist plots before cycles and after 1000 cycles with the insert of equivalent circuit model.


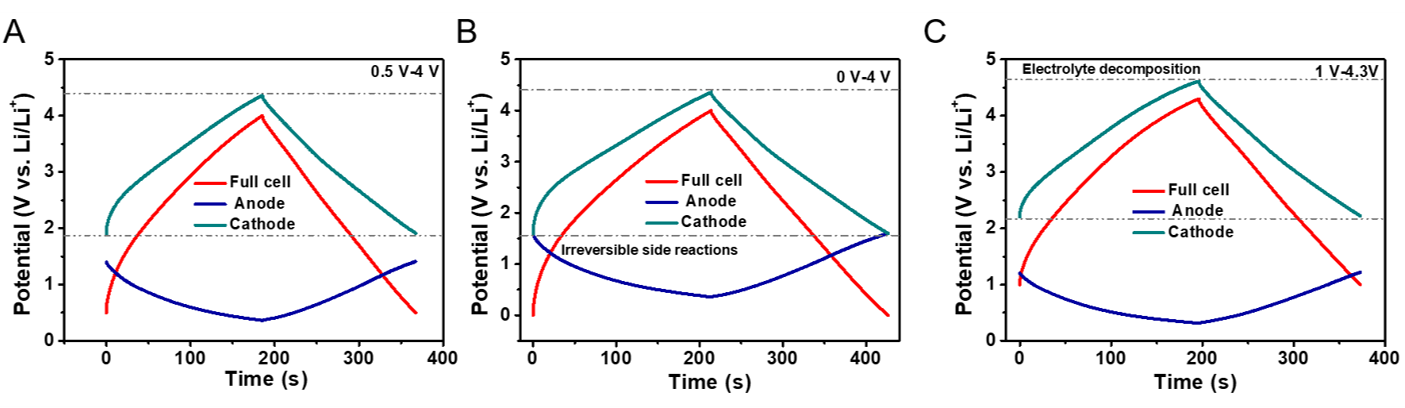


**FIGURE S9.** Galvanostatic charge-discharge profiles at a working voltage window of **(A)** 0.5-4 V, **(B)** 0-4 V and **(C)** 1-4.3 V in a three-electrode configuration.


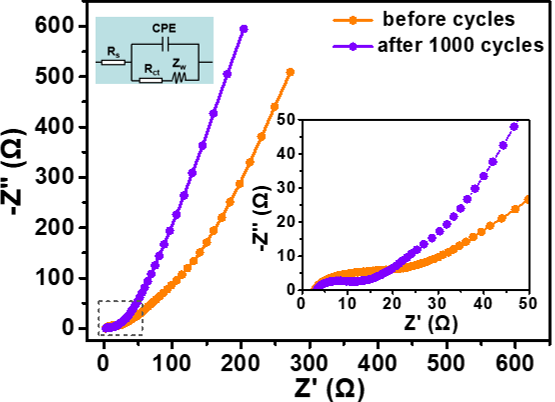


**FIGURE S10.** Nyquist plots of the NPHCS@PPy//NPC LIC device before cycles and after 1000 cycles with the insert of equivalent circuit model.

**TABLE S1.** Comparison of the electrochemical performance between the present NPHCS@PPy//NPC LIC and previously reported LICs.

| LIC device (anode//cathode) | Voltage window (V) | Energy density and power density | Capacity retention | Ref. |
| --- | --- | --- | --- | --- |
| N-doped porous carbon (NPC)//NPC | 0-4.5 | 117 Wh kg^-1^@500 W kg^-1^, 88 Wh kg^-1^@10 kW kg^-1^ | 81%，8000 cycles, 2 A g^-1^ | (Zou et al., 2020) |
| High-defect mesoporous carbon (HDMPC)//HDMPC | 1.0-4.0 | 106 Wh kg^-1^@500 W kg^-1^, 10 Wh kg^-1^ @89 kW kg^-1^ | 88%，8000 cycles, 2 A g^-1^ | (Niu et al., 2018) |
| Fe_3_O_4_@C//  CNT@PPy | 1.0-4.0 | 101 Wh kg^-1^@2709 W kg^-1^, 70 Wh kg^-1^@17186 W kg^-1^ | 80%, 2000 cycles, 10 A g^-1^ | (Han et al., 2019) |
| MnO@C//Porous carbon | 0.1-4.0 | 118 Wh kg^-1^@410 W kg^-1^, 28 Wh kg^-1^ @10250 W kg^-1^ | 76%, 3000 cycles, 1 A g^-1^ | (Yan et al., 2018) |
| ZnSe@CoSe_2_@C@N-doped carbon //AC | 0-4.0 | 135 Wh kg^-1^@342 W kg^-1^, 73 Wh kg^-1^ @6670 W kg^-1^ | 94%，5000 cycles, 1 A g^-1^ | (Chen et al., 2020) |
| Pre-lithiated graphene//AC | 2.0-4.0 | 62 Wh kg^-1^@222 W kg^-1^ | 74%, 300 cycles, 0.4 A g^-1^ | (Ren et al., 2014) |
| TiNb_2_O_7_@C//  Carbon fibers | 0.8-3.2 | 110 Wh kg^-1^@100 W kg^-1^, 20 Wh kg^-1^ @5564 W kg^-1^ | 77%，1500 cycles, 0.2 A g^-1^ | (Wang and Shen, 2015) |
| Porous carbon frameworks (PCF)//PCF | 1.0-4.2 | 112 Wh kg^-1^@260 W kg^-1^, 48 Wh kg^-1^@52 kW kg^-1^ | 70%，9000 cycles, 1 A g^-1^ | (Qian et al., 2021) |
| Carbonized NiCo_2_O_4_// Vertically aligned carbon nanoflakes | 1.0-4.0 | 137 Wh kg^-1^@200 W kg^-1^, 26 Wh kg^-1^ @40 kW kg^-1^ | 90%，9000 cycles, 4 A g^-1^ | (Cheng et al., 2019) |
| MnNCN//AC | 0.1-4.0 | 103 Wh kg^-1^ @ ~150 W kg^-1^ | No degradation, 5000 cycles, 4 A g^-1^ | (Liu et al., 2017) |
| Co_3_ZnC@N-doped carbon//Mesoporous carbon | 1.0-4.5 | 141 Wh kg^-1^@275 W kg^-1^, 15 Wh kg^-1^@10300 W kg^-1^ | 80%, 1000 cycles, 1 A g^-1^ | (Zhu et al., 2018) |
| NPHCS@PPy//NPC | 0.5-4.0 | 145 Wh kg^-1^@225 W kg^-1^, 59 Wh kg^-1^@22500 W kg^-1^ | 92%, 7500 cycles, 1 A g^-1^ | This work |

**References**

Chen, D., Sun, S., Yu, G., Qin, L., Wang, W., Jiang, M., et al. (2020). In -situ thermally fabricated porous and heterogeneous yolk-shell selenides wrapped in carbon as anode for high-performance hybrid lithium -ion capacitors. *Carbon* 166**,** 91-100. doi: 10.1016/j.carbon.2020.05.008

Cheng, C.-F., Li, X., Liu, K., Zou, F., Tung, W.-Y., Huang, Y.-F., et al. (2019). A high-performance lithium-ion capacitor with carbonized NiCo_2_O_4_ anode and vertically-aligned carbon nanoflakes cathode. *Energy Storage Mater.* 22**,** 265-274. doi: https://doi.org/10.1016/j.ensm.2019.07.034

Han, C., Shi, R., Zhou, D., Li, H., Xu, L., Zhang, T., et al. (2019). High-energy and high-power nonaqueous lithium-ion capacitors based on polypyrrole/carbon nanotube composites as pseudocapacitive cathodes. *ACS Appl. Mater. Interfaces* 11**,** 15646-15655. doi: 10.1021/acsami.9b02781

Liu, C., Zhang, C., Fu, H., Nan, X., and Cao, G. (2017). Exploiting high-performance anode through tuning the character of chemical bonds for Li-ion batteries and capacitors. *Adv. Energy Mater.* 7, 1601127. doi: 10.1002/aenm.201601127

Liu, C., Zhang, C., Yin, G., Zhang, T., Wang, W., Ou, G., et al. (2021). A three-dimensional branched TiO_2_ photoanode with an ultrathin Al_2_O_3_ passivation layer and a NiOOH cocatalyst toward photoelectrochemical water oxidation. *ACS Appl. Mater. Interfaces* 13**,** 13301-13310. doi: 10.1021/acsami.1c00948

Niu, J., Shao, R., Liu, M., Liang, J., Zhang, Z., Dou, M., et al. (2018). Porous carbon electrodes with battery-capacitive storage features for high performance Li-ion capacitors. *Energy Storage Mater.* 12**,** 145-152. doi: 10.1016/j.ensm.2017.12.012

Pu, Z., Zhao, J., Amiinu, I.S., Li, W., Wang, M., He, D., et al. (2019). A universal synthesis strategy for P-rich noble metal diphosphide-based electrocatalysts for the hydrogen evolution reaction. *Energy Environ. Sci.* 12**,** 952-957. doi: 10.1039/c9ee00197b

Qian, T., Huang, Y., Zhang, M., Xia, Z., Liu, H., Guan, L., et al. (2021). Non-corrosive and low-cost synthesis of hierarchically porous carbon frameworks for high-performance lithium-ion capacitors. *Carbon* 173**,** 646-654. doi: 10.1016/j.carbon.2020.11.051

Qin, S., Yao, Y., Qian, M., Yang, Q., Chen, T., Xu, H., et al. (2020). Hierarchical microspheres composed of Mn-doped CoP nanosheets for enhanced oxygen evolution. *ACS Appl. Nano Mater.* 3**,** 10702-10707. doi: 10.1021/acsanm.0c01942

Ren, J.J., Su, L.W., Qin, X., Yang, M., Wei, J.P., Zhou, Z., et al. (2014). Pre-lithiated graphene nanosheets as negative electrode materials for Li-ion capacitors with high power and energy density. *J. Power Sources* 264**,** 108-113. doi: 10.1016/j.jpowsour.2014.04.076

Wang, X., and Shen, G. (2015). Intercalation pseudo-capacitive TiNb_2_O_7_@carbon electrode for high-performance lithium ion hybrid electrochemical supercapacitors with ultrahigh energy density. *Nano Energy* 15**,** 104-115. doi: https://doi.org/10.1016/j.nanoen.2015.04.011

Yan, D., Li, S.-H., Guo, L.-P., Dong, X.-L., Chen, Z.-Y., and Li, W.-C. (2018). Hard@soft integrated morning glory like porous carbon as a cathode for a high-energy lithium ion capacitor. *ACS Appl. Mater. Interfaces* 10**,** 43946-43952. doi: 10.1021/acsami.8b17340

Zhu, G., Chen, T., Wang, L., Ma, L., Hu, Y., Chen, R., et al. (2018). High energy density hybrid lithium-ion capacitor enabled by Co_3_ZnC@N-doped carbon nanopolyhedra anode and microporous carbon cathode. *Energy Storage Mater.* 14**,** 246-252. doi: 10.1016/j.ensm.2018.04.009

Zou, K., Guan, Z., Deng, Y., and Chen, G. (2020). Nitrogen-rich porous carbon in ultra-high yield derived from activation of biomass waste by a novel eutectic salt for high performance Li-ion capacitors. *Carbon* 161**,** 25-35. doi: 10.1016/j.carbon.2020.01.045
